# Approximation by modified Kantorovich–Stancu operators

**DOI:** 10.1186/s13660-018-1939-9

**Published:** 2018-12-14

**Authors:** Adonia-Augustina Opriş

**Affiliations:** Department of Mathematics, Technical University of Cluj-Napoca, Cluj-Napoca, Roumania

**Keywords:** 41A25, 41A36, Stancu operators, Kantorovich operators, Stancu–Kantorovich operators, Voronovskaja-type theorem

## Abstract

In the present paper, we study a new kind of Kantorovich–Stancu type operators. For this modified form, we discuss a uniform convergence estimate. Some Voronovskaja-type theorems are given.

## Introduction

Let $0 \le \alpha \le \beta$ and $m \in N$. In [15], D.D. Stancu introduced the linear positive operators

$$P_{m}^{ ( \alpha,\beta )}:C \bigl( [0,1] \bigr) \to C \bigl( [0,1] \bigr) $$

defined by

1.1 $$ P_{m}^{ ( \alpha,\beta )} ( f;x ) = \sum_{k = 0}^{m}p_{m,k} ( x ) f \biggl( \frac{k + \alpha}{m + \beta} \biggr), $$

where

$$p_{m,k} ( x ) = \begin{pmatrix} m \\ k \end{pmatrix} x^{k} ( 1 - x )^{m - k} $$

are the fundamental Bernstein polynomials [3].

When $\alpha = \beta = 0$,

$$P_{m}^{ ( 0,0 )} ( f;x ) = B_{m} ( f;x ) $$

is the classical Bernstein operator.

L.V. Kantorovich [8] introduced the linear positive operators

$$K_{m}:L_{1} \bigl( [0,1] \bigr) \to C \bigl( [0,1] \bigr) $$

defined for any nonnegative integer *m* by

1.2 $$ K_{m} ( f;x ) = ( m + 1 )\sum_{k = 0}^{m} p_{m,k} ( x ) \int_{\frac{k}{m + 1}}^{\frac{k + 1}{m + 1}} f ( s )\,ds. $$

By combining (1.1) and (1.2), D. Bărbosu [2] introduced

$$K_{m}^{ ( \alpha,\beta )}:L_{1} \bigl( [0,1] \bigr) \to C \bigl( [0,1] \bigr) $$

defined for any $m \in N$ by

1.3 $$ K_{m}^{ ( \alpha,\beta )} ( f;x ) = ( m + \beta + 1 )\sum _{k = 0}^{m} p_{m,k} ( x ) \int_{\frac{k + \alpha}{m + \beta + 1}}^{\frac{k + \alpha + 1}{m + \beta + 1}} f ( s )\,ds. $$

$K_{m}^{ ( \alpha,\beta )}$ are linear positive operators called Kantorovich–Stancu operators.

In recent years, Bernstein–Kantorovich–Stancu operators have been modified and studied by many mathematicians. For instance, in [4] Cai et al. defined a new type *λ*-Bernstein operators, and a Kantorovich variant of the modified Bernstein operators was introduced and studied in [7]. In the last three years, Mursaleen et al. investigated several approximation properties for a Kantorovich type generalization of *q*-Bernstein–Stancu operators in [14], applied ($p,q$)-calculus in approximation theory, and constructed the ($p,q$)-analogue of Bernstein operators [12], ($p,q$)-Bernstein–Kantorovich operators [13], and a Kantorovich variant of ($p,q$)-Szász–Mirakjan operators [11]. Also, in [1] Ansari and Karaisa introduced and studied Chlodowsky variant of ($p,q$)-Bernstein operators.

H. Khosravian-Arab, M. Delghan, and M.R. Eslahchi introduced in [9] the following operators:

1.4 $$ B_{m}^{M,1} ( f;x ) = \sum_{k = 0}^{m} p_{m,k}^{M,1} ( x )f \biggl( \frac{k}{m} \biggr), $$

where

1.5 $$ p_{m,k}^{M,1} ( x ) = a ( x;m )p_{m - 1,k} ( x ) + a ( 1 - x;m )p_{m - 1,k - 1} ( x ),\quad x \in [0,1] $$

and

1.6 $$ a ( x;m ) = a_{1} ( m )x + a_{0} ( m ),\quad m = 0,1, \ldots . $$

Here, $a_{0} ( m )$ and $a_{1} ( m )$ are two unknown sequences which are determined in an appropriate way. Note that, for $a_{0} ( m ) = 1$ and $a_{1} ( m ) = - 1$, (1.5) becomes the well-known identity for the fundamental Bernstein polynomials

$$p_{m,k} ( x ) = ( 1 - x )p_{m - 1,k} ( x ) + xp_{m - 1,k - 1} ( x ),\quad 0 < k < m. $$

From (1.5), the operators (1.4) become

$$\begin{aligned} B_{m}^{M,1} ( f;x ) =& a ( x;m )\sum _{k = 0}^{m} p_{m - 1,k} ( x )f \biggl( \frac{k}{m} \biggr) + a ( 1 - x;m )\sum_{k = 0}^{m} p_{m - 1,k - 1} ( x )f \biggl( \frac{k}{m} \biggr) \\ =& a ( x;m )P_{m - 1}^{ ( 0,1 )} ( f;x ) + a ( 1 - x;m )P_{m - 1}^{ ( 1,1 )} ( f;x ). \end{aligned}$$

We try to extend some results to the Kantorovich–Stancu operators considering the operators denoted by

1.7 $$ \overline{K}_{m}^{ ( \alpha,\beta )} ( f;x ) = ( m + \beta + 1 )\sum _{k = 0}^{m} p_{m,k}^{M,1} ( x ) \int_{\frac{k + \alpha}{m + \beta + 1}}^{\frac{k + \alpha + 1}{m + \beta + 1}} f ( s )\,ds,\quad m \in N, x \in [0,1]. $$

## Auxiliary results

### Lemma 2.1

*For* $p \in N^{ *}$, *we have*

$$\begin{aligned} &\mathrm{(i)}\quad P_{m}^{ ( \alpha,\beta )} \bigl( ( t - x )^{p};x \bigr) = \sum_{i = 0}^{p} \begin{pmatrix} p \\ i \end{pmatrix} \frac{1}{ ( m + \beta )^{p - i}}B_{m} \bigl( ( \alpha - t\beta )^{p - i} ( t - x )^{i};x \bigr); \\ &\mathrm{(ii)}\quad K_{m}^{ ( \alpha,\beta )} \bigl( ( t - x )^{p};x \bigr) = \frac{1}{p + 1}\sum_{i = 1}^{p + 1} \begin{pmatrix} p + 1 \\ i \end{pmatrix} \frac{1}{ ( m + \beta + 1 )^{i - 1}}P_{m}^{ ( \alpha,\beta + 1 )} \bigl( ( t - x )^{p + 1 - i};x \bigr). \end{aligned}$$

### Proof

(i)

$$\begin{aligned} P_{m}^{ ( \alpha,\beta )} \bigl( ( t - x )^{p};x \bigr) &= \sum _{k = 0}^{m} p_{m,k} ( x ) \biggl( \frac{k + \alpha}{m + \beta} - x \biggr)^{p} = \sum_{k = 0}^{m} p_{m,k} ( x ) \biggl( \frac{k}{m} - x + \frac{m\alpha - k\beta}{m ( m + \beta )} \biggr)^{p} \\ &= \sum_{k = 0}^{m} p_{m,k} ( x ) \left ( \sum_{i = 0}^{p} \begin{pmatrix} p \\ i \end{pmatrix} \biggl( \frac{k}{m} - x \biggr)^{i} \biggl( \frac{m\alpha - k\beta}{m ( m + \beta )} \biggr)^{p - i} \right ) \\ &= \sum_{k = 0}^{m} p_{m,k} ( x ) \left ( \sum_{i = 0}^{p} \begin{pmatrix} p \\ i \end{pmatrix} \frac{1}{ ( m + \beta )^{p - i}} \biggl( \alpha - \frac{k}{m}\beta \biggr)^{p - i} \biggl( \frac{k}{m} - x \biggr)^{i} \right ) \\ &= \sum_{i = 0}^{p} \begin{pmatrix} p \\ i \end{pmatrix} \frac{1}{ ( m + \beta )^{p - i}} \Biggl( \sum_{k = 0}^{m} p_{m,k} ( x ) \biggl( \alpha - \frac{k}{m}\beta \biggr)^{p - i} \biggl( \frac{k}{m} - x \biggr)^{i} \Biggr) \\ &= \sum_{i = 0}^{p} \begin{pmatrix} p \\ i \end{pmatrix} \frac{1}{ ( m + \beta )^{p - i}}B_{m} \bigl( ( \alpha - t\beta )^{p - i} ( t - x )^{i};x \bigr). \end{aligned}$$

(ii) We have that

$$\begin{aligned} &K_{m}^{ ( \alpha,\beta )} \bigl( ( t - x )^{p};x \bigr)\\ &\quad = \frac{m + \beta + 1}{p + 1}\sum_{k = 0}^{m} p_{m,k} ( x ) \biggl[ \biggl( \frac{k + \alpha}{m + \beta + 1} - x + \frac{1}{m + \beta + 1} \biggr)^{p + 1} - \biggl( \frac{k + \alpha}{m + \beta + 1} - x \biggr)^{p + 1} \biggr] \\ &\quad = \frac{m + \beta + 1}{p + 1}\sum_{k = 0}^{m} p_{m,k} ( x )\left ( \sum_{i = 1}^{p + 1} \begin{pmatrix} p + 1 \\ i \end{pmatrix} \biggl( \frac{1}{m + \beta + 1} \biggr)^{i} \biggl( \frac{k + \alpha}{m + \beta + 1} - x \biggr)^{p + 1 - i} \right ) \\ &\quad = \frac{1}{p + 1}\sum_{i = 1}^{p + 1} \begin{pmatrix} p + 1 \\ i \end{pmatrix}\frac{1}{ ( m + \beta + 1 )^{i - 1}} \Biggl( \sum_{k = 0}^{m} p_{m,k} ( x ) \biggl( \frac{k + \alpha}{m + \beta + 1} - x \biggr)^{p + 1 - i} \Biggr) \\ &\quad = \frac{1}{p + 1}\sum_{i = 1}^{p + 1} \begin{pmatrix} p + 1 \\ i \end{pmatrix}\frac{1}{ ( m + \beta + 1 )^{i - 1}} P_{m}^{ ( \alpha,\beta + 1 )} \bigl( ( t - x )^{p + 1 - i};x \bigr). \end{aligned}$$

 □

### Corollary 2.2

*For any* $p \in N^{ *}$, *there exists a constant* $C(p)$, *independent of* *m* *and* *x*, *such that*

2.1 $$ \bigl\vert K_{m}^{ ( \alpha,\beta )} \bigl( ( t - x )^{p};x \bigr) \bigr\vert \le C ( p ) \biggl( \frac{x ( 1 - x )}{m} \biggr)^{\frac{p}{2}} + O \biggl( \frac{1}{m^{p}} \biggr) $$

*for every* $x \in [0,1]$.

### Proof

First we have

2.2 $$ \bigl\vert P_{m}^{ ( \alpha,\beta )} \bigl( ( t - x )^{p};x \bigr) \bigr\vert \le \sum_{i = 0}^{p} \begin{pmatrix} p \\ i \end{pmatrix} \biggl( \frac{M}{m + \beta} \biggr)^{p - i}B_{m} \bigl( \vert t - x \vert ^{i};x \bigr), $$

where $M = \max \{ \alpha,\beta - \alpha \}$ for $x \in [0,1 ]$.

The following inequality

2.3 $$ B_{m} \bigl( \vert t - x \vert ^{i};x \bigr) \le c ( i )\sqrt{ \biggl( \frac{X}{m} \biggr)^{i}}, $$

where $c ( i )$ is a constant independent of *m*, can be found in [16] for $mX \ge 1$, $X = x ( 1 - x )$ and in [5] for $mX < 1$.

Taking $c ( p ) = \max_{i = \overline{0,p}}c ( i )$ in (2.3), by (2.2) it follows

2.4 $$\begin{aligned} \bigl\vert P_{m}^{ ( \alpha,\beta )} \bigl( ( t - x )^{p};x \bigr) \bigr\vert \le& c ( p )\sum_{i = 0}^{p} \begin{pmatrix} p \\ i \end{pmatrix} \sqrt{ \biggl( \frac{x ( 1 - x )}{m} \biggr)^{i}} \biggl( \frac{M}{m + \beta} \biggr)^{p - i} \\ \le& c ( p ) \biggl( \sqrt{ \biggl( \frac{x ( 1 - x )}{m} \biggr)} + \frac{M}{m + \beta} \biggr)^{p} \\ \le& 2^{p - 1}c ( p ) \biggl( \sqrt{ \biggl( \frac{x ( 1 - x )}{m} \biggr)}^{p} + \biggl( \frac{M}{m + \beta} \biggr)^{p} \biggr) \\ \le& C ( p ) \biggl( \frac{x ( 1 - x )}{m} \biggr)^{\frac{p}{2}} + O \biggl( \frac{1}{m^{p}} \biggr). \end{aligned}$$

From (2.4) and Lemma 2.1, we obtain estimate (2.1). □

The first four central moments for $K_{m}^{ ( \alpha,\beta )}$ are as follows:

$$\begin{aligned} &K_{m}^{ ( \alpha,\beta )} ( t - x;x ) = - \frac{\beta + 1}{m + \beta + 1}x + \frac{2\alpha + 1}{2 ( m + \beta + 1 )}, \\ & \begin{aligned} K_{m}^{ ( \alpha,\beta )} \bigl( ( t - x )^{2};x \bigr) = {}&\frac{m - ( 2\alpha + 1 ) ( \beta + 1 )}{ ( m + \beta + 1 )^{2}}x ( 1 - x ) \\ &{}+ \frac{ ( \beta + 1 ) ( \beta - 2\alpha )}{ ( m + \beta + 1 )^{2}}x^{2} + \frac{3\alpha^{2} + 3\alpha + 1}{3 ( m + \beta + 1 )^{2}}, \end{aligned} \\ & \begin{aligned} K_{m}^{ ( \alpha,\beta )} \bigl( ( t - x )^{3};x \bigr) ={}& {-} \frac{ ( 3\beta + 5 )m - ( \beta + 1 )^{3}}{ ( m + \beta + 1 )^{3}}x^{2} ( 1 - x ) \\ &{}+ \frac{ ( 12\alpha + 10 )m - 6 ( 2\alpha + 1 ) ( \beta + 1 )^{2} + 4 ( \beta + 1 )^{3}}{4 ( m + \beta + 1 )^{3}}x ( 1 - x ) \\ &{}- \frac{4 ( 3\alpha^{2} + 3\alpha + 1 ) ( \beta + 1 ) - 6 ( 2\alpha + 1 ) ( \beta + 1 )^{2} + 4 ( \beta + 1 )^{3}}{4 ( m + \beta + 1 )^{3}}x \\ &{}+ \frac{4\alpha^{3} + 6\alpha^{2} + 4\alpha + 1}{4 ( m + \beta + 1 )^{3}}, \end{aligned} \\ & K_{m}^{ ( \alpha,\beta )} \bigl( ( t - x )^{4};x \bigr) \\ &\quad = \frac{3m^{2} - 2 ( 3 + 4 ( \beta + 1 ) + 3 ( \beta + 1 )^{2} )m}{ ( m + \beta + 1 )^{4}} \bigl( x ( 1 - x ) \bigr)^{2} \\ &\qquad {}- \frac{ [ 4 ( 2\alpha + 1 ) + 2 ( 6\alpha + 1 ) ( \beta + 1 ) - 6 ( \beta + 1 )^{2} ]m - 2 ( 2\alpha + 1 ) ( \beta + 1 )^{3}}{ ( m + \beta + 1 )^{4}}x^{2} ( 1 - x ) \\ &\qquad {}+ \frac{ ( 6\alpha^{2} + 10\alpha + 5 )m + 2 ( 2\alpha + 1 ) ( \beta + 1 )^{3} - 2 ( 3\alpha^{2} + 3\alpha + 1 ) ( \beta + 1 )^{2}}{ ( m + \beta + 1 )^{4}}x ( 1 - x ) \\ &\qquad {}- \frac{2 ( 2\alpha + 1 ) ( \beta + 1 )^{3} - 2 ( 3\alpha^{2} + 3\alpha + 1 ) ( \beta + 1 )^{2} + ( 4\alpha^{3} + 6\alpha^{2} + 4\alpha + 1 ) ( \beta + 1 )}{ ( m + \beta + 1 )^{4}}x \\ &\qquad {}+ \frac{5\alpha^{4} + 10\alpha^{3} + 10\alpha^{2} + 5\alpha + 1}{5 ( m + \beta + 1 )^{4}}. \end{aligned}$$

### Remark 2.3

Using the results obtained by Gavrea and Ivan ([5], Theorem 14, Theorem 15, Remark 16), it is straightforward to give the following estimates:

(i) For any $p \ge 4$ and $x \in ( 0,1 )$, there exists a constant $A ( p )$ independent of *m* and *x* such that
  2.5 $$ \frac{K_{m}^{ ( \alpha,\beta )} ( \vert t - x \vert ^{p + 1};x )}{K_{m}^{ ( \alpha,\beta )} ( \vert t - x \vert ^{p};x )} \le \frac{A ( p )}{\sqrt{m}},\quad m \ge 5. $$
(ii) For any $p \ge 1$ and $x \in ( 0,1 )$, there exists a positive constant $B ( p )$ independent of *m* and *x* such that
  2.6 $$ \biggl\Vert \frac{K_{m}^{ ( \alpha,\beta )} ( \vert t - x \vert ^{p + 1};x )}{K_{m}^{ ( \alpha,\beta )} ( \vert t - x \vert ^{p};x )} \biggr\Vert \ge \frac{B ( p )}{\sqrt{m}}, $$
  where $\Vert \cdot \Vert $ is the uniform norm on $[0,1]$.
(iii) From (i) and (ii) it follows
  2.7 $$ \biggl\Vert \frac{K_{m}^{ ( \alpha,\beta )} ( \vert t - x \vert ^{p + 1};x )}{K_{m}^{ ( \alpha,\beta )} ( \vert t - x \vert ^{p};x )} \biggr\Vert = O \biggl( \frac{1}{\sqrt{m}} \biggr). $$

### Remark 2.4

From Mamedov’s theorem [10] it follows that:

If $p \in N^{ *} $ is even and $f \in C^{p} ( [0,1] )$, for any $x \in [0,1]$, we have that

2.8 $$ \lim_{m \to \infty} \frac{1}{K_{m}^{ ( \alpha,\beta )} ( ( t - x )^{p};x )} \Biggl( K_{m}^{ ( \alpha,\beta )} ( f;x ) - f ( x ) - \sum _{i = 1}^{p} K_{m}^{ ( \alpha,\beta )} \bigl( ( t - x )^{i};x \bigr) \frac{f^{ ( i )} ( x )}{i!} \Biggr) = 0. $$

## Modified Kantorovich–Stancu operators

Now, we modify the Kantorovich–Stancu operator as follows:

3.1 $$ \overline{K}_{m}^{ ( \alpha,\beta )} ( f;x ) = a ( x;m )K_{m - 1}^{ ( \alpha,\beta + 1 )} ( f;x ) + a ( 1 - x;m )K_{m - 1}^{ ( \alpha + 1,\beta + 1 )} ( f;x ). $$

### Lemma 3.1

*The moments* $\overline{K}_{m}^{ ( \alpha,\beta )} ( t^{i};x )$, $i = 0,1,2$, *are given by*

$$\begin{aligned} &\overline{K}_{m}^{ ( \alpha,\beta )} ( 1;x ) = 2a_{0} ( m ) + a_{1} ( m ), \\ &\begin{aligned} \overline{K}_{m}^{ ( \alpha,\beta )} ( t;x ) ={}& \frac{ ( 2a_{0} ( m ) + a_{1} ( m ) )m}{m + \beta + 1}x \\ &{}+ \frac{4 ( \alpha + 1 )a_{0} ( m ) + ( 2\alpha + 3 )a_{1} ( m ) - 4 ( a_{0} ( m ) + a_{1} ( m ) )x}{2 ( m + \beta + 1 )}, \end{aligned} \\ &\begin{aligned} \overline{K}_{m}^{ ( \alpha,\beta )} \bigl( t^{2};x \bigr) ={}& \frac{ ( 2a_{0} ( m ) + a_{1} ( m ) )m^{2}}{ ( m + \beta + 1 )^{2}}x^{2} \\ &{}+ \frac{ [ 2 ( ( 2\alpha + 3 )a_{0} ( m ) + ( \alpha + 2 )a_{1} ( m ) )x - ( 6a_{0} ( m ) + 5a_{1} ( m ) )x^{2} ]m}{ ( m + \beta + 1 )^{2}} \\ &{}+ \frac{12 ( a_{0} ( m ) + a_{1} ( m ) )x^{2} - 6 ( a_{0} ( m ) + a_{1} ( m ) ) ( 2\alpha + 3 )x}{3 ( m + \beta + 1 )^{2}} \\ &{}+ \frac{2 ( 3\alpha^{2} + 6\alpha + 4 )a_{0} ( m ) + ( 3\alpha^{2} + 9\alpha + 7 )a_{1} ( m )}{3 ( m + \beta + 1 )^{2}}. \end{aligned} \end{aligned}$$

### Lemma 3.2

*The central moments of the operators* $\overline{K}_{m}^{ ( \alpha,\beta )}$, $\overline{K}_{m}^{ ( \alpha,\beta )} ( ( t - x )^{i};x )$, $i = 1,2,3,4$, *are given by*

$$\begin{aligned} &\overline{K}_{m}^{ ( \alpha,\beta )} ( t - x;x ) = - \frac{2 ( \beta + 2 )a_{0} ( m ) + ( \beta + 3 )a_{1} ( m )}{m + \beta + 1}x + \frac{4 ( \alpha + 1 )a_{0} ( m ) + ( 2\alpha + 3 )a_{1} ( m )}{2 ( m + \beta + 1 )}, \\ &\overline{K}_{m}^{ ( \alpha,\beta )} \bigl( ( t - x )^{2};x \bigr) = \frac{ ( 2a_{0} ( m ) + a_{1} ( m ) )m}{ ( m + \beta + 1 )^{2}}x ( 1 - x ) + O \biggl( \frac{1}{m^{2}} \biggr), \\ &\begin{aligned} \overline{K}_{m}^{ ( \alpha,\beta )} \bigl( ( t - x )^{3};x \bigr) ={}& {-} \frac{ ( ( 3\beta + 8 ) ( 2a_{0} ( m ) + a_{1} ( m ) ) + 3a_{1} ( m ) )m}{ ( m + \beta + 1 )^{3}}x^{2} ( 1 - x ) \\ &{}+ \frac{ ( ( 12\alpha + 10 ) ( 2a_{0} ( m ) + a_{1} ( m ) ) + 12 ( a_{0} ( m ) + a_{1} ( m ) ) )m}{4 ( m + \beta + 1 )^{3}}x ( 1 - x ) \\ &{}+ O \biggl( \frac{1}{m^{3}} \biggr), \end{aligned} \\ &\overline{K}_{m}^{ ( \alpha,\beta )} \bigl( ( t - x )^{4};x \bigr) = \frac{3 ( 2a_{0} ( m ) + a_{1} ( m ) )m^{2}}{ ( m + \beta + 1 )^{4}} \bigl( x ( 1 - x ) \bigr)^{2} + O \biggl( \frac{1}{m^{4}} \biggr). \end{aligned}$$

We will study the uniform convergence of the sequence $( \overline{K}_{m}^{ ( \alpha,\beta )}f )_{m \in N}$ for the case

3.2 $$ 2a_{0} ( m ) + a_{1} ( m ) = 1. $$

We observe that (3.2) implies $\overline{K}_{m}^{ ( \alpha,\beta )} ( 1;x ) = 1$.

We are interested in the following cases:

Case 1:

3.3 $$ a_{0} ( m ) \ge 0\quad \mbox{and}\quad a_{0} ( m ) + a_{1} ( m ) \ge 0. $$

Case 2:

3.4 $$ a_{1} ( m ) < 0\quad \mbox{or}\quad a_{0} ( m ) + a_{1} ( m ) < 0. $$

Combining (3.2) and (3.3), we obtain $a_{0} ( m ) \in [0,1]$ and $a_{1} ( m ) \in [-1,1]$, which implies that the sequences $a_{0} ( m )$ and $a_{1} ( m )$ are bounded. The operators $\overline{K}_{m}^{ ( \alpha,\beta )}$ are bounded and positive.

Combining (3.2) and (3.4), we obtain that $a_{0} ( m ) + a_{1} ( m ) > 1$ if $a_{1} ( m ) < 0$ and $a_{0} ( m ) > 1$ if $a_{0} ( m ) + a_{1} ( m ) < 0$. In these cases, the operators $\overline{K}_{m}^{ ( \alpha,\beta )}$ are not positive.

Remark that, for $\alpha = \beta = 0$ and $a_{0} ( m ) = \frac{3}{2}$, $a_{1} ( m ) = - 2$, we obtain the modified operators introduced and studied in [7].

In order to prove the uniform convergence of the operators $\overline{K}_{m}^{ ( \alpha,\beta )}$, we give the Korovkin theorem:

### Theorem 3.3

([9], Theorem 10)

*Let* $0 < h \in C ( [ a,b ] )$ *be a function and suppose that* $( L_{n} )_{n \ge 1}$ *is a sequence of positive linear operators such that* $\lim_{n \to \infty} L_{n} ( e_{i} ) = he_{i}$, $i = 0,1,2$, *uniformly on* $[ a,b ]$. *Then*, *for a given function* $f \in C ( [ a,b ] )$, *we have* $\lim_{n \to \infty} L_{n} ( f ) = hf$ *uniformly on* $[ a,b ]$.

For the first case, we obtain the following result:

### Theorem 3.4

*Given two sequences* $a_{0} ( m )$ *and* $a_{1} ( m )$ *that satisfy conditions* (3.2) *and* (3.3), *the sequence* $( \overline{K}_{m}^{ ( \alpha,\beta )}f )_{m \in N}$ *converges to* *f*, *uniformly on* $[0,1]$, *for any function* $f \in C ( [0,1] )$.

### Proof

The operator $\overline{K}_{m}^{ ( \alpha,\beta )}f$ is a linear convex combination of positive operators $K_{m - 1}^{ ( \alpha,\beta + 1 )}f$ and $K_{m - 1}^{ ( \alpha + 1,\beta + 1 )}f$. Consequently, the result follows from Theorem 3.3. □

In the second case, we have the following:

### Theorem 3.5

*For any function* $f \in C ( [0,1] )$ *and all bounded sequences* $a_{0} ( m ), a_{1} ( m )$ *that satisfy conditions* (3.2) *and* (3.4), *the sequence* $( \overline{K}_{m}^{ ( \alpha,\beta )}f )_{m \in N}$ *converges to* *f*, *uniformly on* $[0,1]$.

### Proof

$$\begin{aligned} \overline{K}_{m}^{ ( \alpha,\beta )} ( f;x ) ={}& a ( x;m )K_{m - 1}^{ ( \alpha,\beta + 1 )} ( f;x ) + a ( 1 - x;m )K_{m - 1}^{ ( \alpha + 1,\beta + 1 )} ( f;x ) \\ ={}& \bigl( a_{1} ( m )x + a_{0} ( m ) \bigr)K_{m - 1}^{ ( \alpha,\beta + 1 )} ( f;x )\\ &{} + \bigl( - a_{1} ( m )x + a_{0} ( m ) + a_{1} ( m ) \bigr)K_{m - 1}^{ ( \alpha + 1,\beta + 1 )} ( f;x ) \\ ={}& \bigl[ a_{0} ( m )K_{m - 1}^{ ( \alpha,\beta + 1 )} ( f;x ) + \bigl( a_{0} ( m ) - a_{1} ( m )x \bigr)K_{m - 1}^{ ( \alpha + 1,\beta + 1 )} ( f;x ) \bigr] \\ &{}- \bigl[ - a_{1} ( m )xK_{m - 1}^{ ( \alpha,\beta + 1 )} ( f;x ) - a_{1} ( m )K_{m - 1}^{ ( \alpha + 1,\beta + 1 )} ( f;x ) \bigr]. \end{aligned}$$

Taking

$$\overline{K}_{m,1}^{ ( \alpha,\beta )} ( f;x ) = a_{0} ( m )K_{m - 1}^{ ( \alpha,\beta + 1 )} ( f;x ) + \bigl( a_{0} ( m ) - a_{1} ( m )x \bigr)K_{m - 1}^{ ( \alpha + 1,\beta + 1 )} ( f;x ) $$

and

$$\overline{K}_{m,2}^{ ( \alpha,\beta )} ( f;x ) = - a_{1} ( m )xK_{m - 1}^{ ( \alpha,\beta + 1 )} ( f;x ) - a_{1} ( m )K_{m - 1}^{ ( \alpha + 1,\beta + 1 )} ( f;x ), $$

we have

3.5 $$ \overline{K}_{m}^{ ( \alpha,\beta )} ( f;x ) = \overline{K}_{m,1}^{ ( \alpha,\beta )} ( f;x ) - \overline{K}_{m,2}^{ ( \alpha,\beta )} ( f;x ). $$

Using the remarks for case 2, it follows that the operators $\overline{K}_{m,1}^{ ( \alpha,\beta )}$ and $\overline{K}_{m,2}^{ ( \alpha,\beta )}$ are positive. According to Theorems 3.3 and 3.4, we obtain that

$$\begin{aligned} \lim_{m \to \infty} \bigl( \overline{K}_{m}^{ ( \alpha,\beta )}f \bigr) ( x ) &= \lim_{m \to \infty} \bigl( \overline{K}_{m,1}^{ ( \alpha,\beta )}f \bigr) ( x ) - \lim_{m \to \infty} \bigl( \overline{K}_{m,2}^{ ( \alpha,\beta )}f \bigr) ( x ) \\ &= ( 2l_{0} - l_{1}x )f ( x ) + l_{1} ( 1 + x )f ( x ) \\ &= ( 2l_{0} + l_{1} )f ( x ) = f ( x ), \end{aligned}$$

where $l_{i} = \lim_{m \to \infty} a_{i} ( m )$, $i = 0,1$. □

The following theorems are Voronovskaja-type results for the operators $\overline{K}_{m}^{ ( \alpha,\beta )}$.

### Theorem 3.6

*Let* $a_{0} ( m )$, $a_{1} ( m )$ *be two convergent sequences that verify conditions* (3.2) *and* (3.3) *and* $l_{i} = \lim_{m \to \infty} a_{i} ( m )$, $i = 0,1$. *If* $f \in C^{2} ( [0,1] )$, *then*

3.6 $$\begin{aligned} \lim_{m \to \infty} m \bigl( \overline{K}_{m}^{ ( \alpha,\beta )} ( f;x ) - f ( x ) \bigr) =& \biggl( \bigl( \alpha + 1 - ( \beta + 2 )x \bigr) + \frac{ ( 1 - 2x )l_{1}}{2} \biggr)f' ( x ) \\ &{}+ \frac{1}{2}x ( 1 - x )f'' ( x ) \end{aligned}$$

*uniformly on* $[0,1]$.

### Proof

Applying Taylor’s formula to the operators $\overline{K}_{m}^{ ( \alpha,\beta )}$, we have

3.7 $$\begin{aligned} \overline{K}_{m}^{ ( \alpha,\beta )} ( f;x ) =& f ( x ) + \frac{1}{1!}\overline{K}_{m}^{ ( \alpha,\beta )} ( t - x;x )f' ( x ) + \frac{1}{2!}\overline{K}_{m}^{ ( \alpha,\beta )} \bigl( ( t - x )^{2};x \bigr)f'' ( x ) \\ &{}+ \overline{K}_{m}^{ ( \alpha,\beta )} \bigl( \rho ( t;x ) ( t - x )^{2};x \bigr), \end{aligned}$$

where $\rho \in C ( [0,1] )$ and $\lim_{t \to x}\rho ( t;x ) = 0$.

It is sufficient to prove that $\lim_{m \to \infty} m\overline{K}_{m}^{ ( \alpha,\beta )} ( \rho ( t;x ) ( t - x )^{2};x ) = 0$ uniformly on $[0,1]$.

Using the Cauchy–Schwarz theorem, we obtain that

$$\overline{K}_{m}^{ ( \alpha,\beta )} \bigl( \rho ( t;x ) ( t - x )^{2};x \bigr) \le \sqrt{\overline{K}_{m}^{ ( \alpha,\beta )} \bigl( \bigl( \rho ( t;x ) \bigr)^{2};x \bigr) \cdot \overline{K}_{m}^{ ( \alpha,\beta )} \bigl( ( t - x )^{4};x \bigr)}. $$

Since $\rho ( x,x ) = 0, \rho^{2} ( \cdot;x ) \in C ( [0,1] )$, by Theorem 3.4, we have

$$\lim_{m \to \infty} \overline{K}_{m}^{ ( \alpha,\beta )} \bigl( \bigl( \rho ( t;x ) \bigr)^{2};x \bigr) = 0, $$

and by Lemma 3.2, we get

$$\lim_{m \to \infty} m^{2}\overline{K}_{m}^{ ( \alpha,\beta )} \bigl( ( t - x )^{4};x \bigr) = 3 \bigl( x ( 1 - x ) \bigr)^{2} $$

uniformly on $[0,1]$. Hence, we obtain the above limit.

Finally, Lemma 3.2 gives us (3.6). □

### Theorem 3.7

*Let* $a_{0} ( m )$, $a_{1} ( m )$ *be two bounded convergent sequences that verify conditions* (3.2) *and* (3.4) *and* $l_{i} = \lim_{m \to \infty} a_{i} ( m )$, $i = 0,1$. *If* $f \in C^{2} ( [0,1] )$, *then*

$$\lim_{m \to \infty} m \bigl( \overline{K}_{m}^{ ( \alpha,\beta )} ( f;x ) - f ( x ) \bigr) = \biggl( \bigl( \alpha + 1 - ( \beta + 2 )x \bigr) + \frac{ ( 1 - 2x )l_{1}}{2} \biggr)f' ( x ) + \frac{1}{2}x ( 1 - x )f^{\prime\prime} ( x ) $$

*uniformly on* $[0,1]$.

### Proof

From (3.5), we have

$$\overline{K}_{m}^{ ( \alpha,\beta )} ( f;x ) = \overline{K}_{m,1}^{ ( \alpha,\beta )} ( f;x ) - \overline{K}_{m,2}^{ ( \alpha,\beta )} ( f;x ), $$

where

$$\overline{K}_{m,1}^{ ( \alpha,\beta )} ( f;x ) = a_{0} ( m )K_{m - 1}^{ ( \alpha,\beta + 1 )} ( f;x ) + \bigl( a_{0} ( m ) - a_{1} ( m )x \bigr)K_{m - 1}^{ ( \alpha + 1,\beta + 1 )} ( f;x ) $$

and

$$\overline{K}_{m,2}^{ ( \alpha,\beta )} ( f;x ) = - a_{1} ( m )xK_{m - 1}^{ ( \alpha,\beta + 1 )} ( f;x ) - a_{1} ( m )K_{m - 1}^{ ( \alpha + 1,\beta + 1 )} ( f;x ). $$

Applying Theorem 3.6 to the operators $\overline{K}_{m,2}^{ ( \alpha,\beta )}$ and $\overline{K}_{m,1}^{ ( \alpha,\beta )}$, we obtain

$$\begin{aligned} \lim_{m \to \infty} m \bigl( \overline{K}_{m,1}^{ ( \alpha,\beta )} ( f;x ) - f ( x ) \bigr) =& \biggl( - ( 2l_{0} - l_{1}x ) ( \beta + 2 )x + \frac{4 ( \alpha + 1 )l_{0} - ( 2\alpha + 3 )l_{1}x}{2} \biggr)f' ( x ) \\ &{}+ \frac{1}{2} ( 2l_{0} - l_{1}x )x ( 1 - x )f'' ( x ) \end{aligned}$$

and

$$\begin{aligned} \lim_{m \to \infty} m \bigl( \overline{K}_{m,2}^{ ( \alpha,\beta )} ( f;x ) - f ( x ) \bigr) =& \biggl( l_{1} ( x + 1 ) ( \beta + 2 )x - \frac{ ( 2\alpha + 1 )x + ( 2\alpha + 3 )}{2}l_{1} \biggr)f' ( x ) \\ &{}- \frac{1}{2}l_{1} ( x + 1 )x ( 1 - x )f'' ( x ) \end{aligned}$$

uniformly on $[0,1]$.

Combining these two results, the proof is finished. □

In what follows, we will denote by $\omega ( f; \cdot )$ the first order modulus of continuity of the function *f*

$$\omega ( f;\delta ) = \sup \bigl\{ \bigl\vert f \bigl( x' \bigr) - f \bigl( x^{\prime\prime} \bigr) \bigr\vert |x',x^{\prime\prime} \in I, \bigl\vert x' - x^{\prime\prime} \bigr\vert \le \delta \bigr\} ,\quad \mbox{where } I = [0,1], f:I \to R. $$

### Theorem 3.8

*Let* $a_{0} ( m ), a_{1} ( m )$ *be two bounded sequences that verify* (3.2). *If* $f ( x )$ *is bounded for* $x \in [0,1]$, *then*

3.8 $$ \bigl\Vert \overline{K}_{m}^{ ( \alpha,\beta )}f - f \bigr\Vert \le \frac{3}{2} \bigl( 3 \bigl\vert a_{1} ( m ) \bigr\vert + 1 \bigr)\omega \biggl( f;\frac{1}{\sqrt{m + \beta + 1}} \biggr), $$

*where* $\Vert \cdot \Vert $ *is the uniform norm on* $[0,1]$.

### Proof

By (3.1), we have that

3.9 $$\begin{aligned} \bigl\vert \overline{K}_{m}^{ ( \alpha,\beta )} ( f;x ) - f ( x ) \bigr\vert \le& \bigl\vert a ( x;m ) \bigr\vert \bigl\vert K_{m - 1}^{ ( \alpha,\beta + 1 )} ( f;x ) - f ( x ) \bigr\vert \\ &{}+ \bigl\vert a ( 1 - x;m ) \bigr\vert \bigl\vert K_{m - 1}^{ ( \alpha + 1,\beta + 1 )} ( f;x ) - f ( x ) \bigr\vert . \end{aligned}$$

We need an upper bound for $a ( x;m )$ and $a ( 1 - x;m )$. Note that this is the same upper bound for both. From (3.2), it follows that

$$\begin{aligned} \bigl\vert a ( x;m ) \bigr\vert =& \bigl\vert a_{1} ( m )x + a_{0} ( m ) \bigr\vert \le \bigl\vert a_{1} ( m ) \bigr\vert + \bigl\vert a_{0} ( m ) \bigr\vert \\ =& \bigl\vert a_{1} ( m ) \bigr\vert + \biggl\vert \frac{1 - a_{1} ( m )}{2} \biggr\vert \le \frac{3 \vert a_{1} ( m ) \vert + 1}{2} \end{aligned}$$

and (3.9) becomes

3.10 $$\begin{aligned} \bigl\vert \overline{K}_{m}^{ ( \alpha,\beta )} ( f;x ) - f ( x ) \bigr\vert \le& \frac{3 \vert a_{1} ( m ) \vert + 1}{2} \bigl[ \bigl\vert K_{m - 1}^{ ( \alpha,\beta + 1 )} ( f;x ) - f ( x ) \bigr\vert \\ &{} + \bigl\vert K_{m - 1}^{ ( \alpha + 1,\beta + 1 )} ( f;x ) - f ( x ) \bigr\vert \bigr]. \end{aligned}$$

By ([2], Theorem 2.6), we have

$$\bigl\vert K_{m - 1}^{ ( \alpha,\beta + 1 )} ( f;x ) - f ( x ) \bigr\vert \le 2\omega \Bigl( f;\sqrt{\delta_{m - 1,1}^{ ( \alpha,\beta + 1 )}} \Bigr) $$

and

$$\bigl\vert K_{m - 1}^{ ( \alpha + 1,\beta + 1 )} ( f;x ) - f ( x ) \bigr\vert \le 2\omega \Bigl( f;\sqrt{\delta_{m - 1,1}^{ ( \alpha + 1,\beta + 1 )}} \Bigr), $$

where

$$\begin{aligned} &\begin{aligned} \delta_{m - 1,1}^{ ( \alpha,\beta + 1 )} ={}& \frac{ ( \beta + 2 )^{2}}{ ( m + \beta + 1 )^{2}} + \frac{ ( 2\alpha + 1 ) ( m - 1 )^{2}}{ ( m + \beta ) ( m + \beta + 1 )^{2}} + \frac{m - 1}{4 ( m + \beta + 1 )^{2}} \\ &{}+ \frac{3\alpha^{2} ( 3m + \beta - 2 ) + ( m + \beta ) ( 1 - 3m - 3\beta )}{3 ( m + \beta ) ( m + \beta + 1 )^{2}}, \end{aligned} \\ &\begin{aligned} \delta_{m - 1,1}^{ ( \alpha + 1,\beta + 1 )} ={}& \frac{ ( \beta + 2 )^{2}}{ ( m + \beta + 1 )^{2}} + \frac{ ( 2\alpha + 3 ) ( m - 1 )^{2}}{ ( m + \beta ) ( m + \beta + 1 )^{2}} + \frac{m - 1}{4 ( m + \beta + 1 )^{2}} \\ &{}+ \frac{3 ( \alpha + 1 )^{2} ( 3m + \beta - 2 ) + ( m + \beta ) ( 1 - 3m - 3\beta )}{3 ( m + \beta ) ( m + \beta + 1 )^{2}}. \end{aligned} \end{aligned}$$

So,

3.11 $$ \bigl\vert \overline{K}_{m}^{ ( \alpha,\beta )} ( f;x ) - f ( x ) \bigr\vert \le \bigl( 3 \bigl\vert a_{1} ( m ) \bigr\vert + 1 \bigr) \Bigl( \omega \Bigl( f;\sqrt{\delta_{m - 1,1}^{ ( \alpha,\beta + 1 )}} \Bigr) + \omega \Bigl( f;\sqrt{\delta_{m - 1,1}^{ ( \alpha + 1,\beta + 1 )}} \Bigr) \Bigr). $$

By using the properties of the first order modulus of continuity together with the above forms of $\delta_{m - 1,1}^{ ( \alpha,\beta + 1 )}$and $\delta_{m - 1,1}^{ ( \alpha + 1,\beta + 1 )}$ in (3.11), we obtain (3.8). □

Assume that $\beta = 2\alpha$, $\overline{K}_{m}^{ ( \alpha,\beta )} ( 1;x ) = 1$ and $\overline{K}_{m}^{ ( \alpha,\beta )} ( t;x ) = x$.

Consequently, we get

$$2a_{0} ( m ) + a_{1} ( m ) = 1\quad \mbox{and}\quad a_{0} ( m ) + a_{1} ( m ) = - \frac{2\alpha + 1}{2}, $$

which implies that

$$a_{0} ( m ) = \frac{2\alpha + 3}{2},\qquad a_{1} ( m ) = - 2 ( \alpha + 1 ) $$

and from (3.4), it follows that the operators $\overline{K}_{m}^{ ( \alpha,\beta )}$ are not positive.

Now, we can formulate a new quantitative Voronovskaja-type result:

### Theorem 3.9

*For* $g \in C^{2} ( [0,1] )$, $x \in [0,1]$ *fixed*, *we have the following estimate*:

3.12 $$ \biggl\vert \overline{K}_{m}^{ ( \alpha,\beta )} ( g;x ) - g ( x ) - \frac{1}{2}\overline{K}_{m}^{ ( \alpha,\beta )} \bigl( ( t - x )^{2};x \bigr)g'' ( x ) \biggr\vert \le C \frac{1}{m}\omega \biggl( g'';\frac{1}{\sqrt{m + \beta + 1}} \biggr), $$

*where* *C* *is a positive constant independent of* *m* *and* *x*.

### Proof

Under the above assumptions, by applying Taylor’s formula to the operators $\overline{K}_{m}^{ ( \alpha,\beta )}$, we have

3.13 $$ \overline{K}_{m}^{ ( \alpha,\beta )} ( g;x ) = g ( x ) + \frac{1}{2!} \overline{K}_{m}^{ ( \alpha,\beta )} \bigl( ( t - x )^{2};x \bigr)g'' ( x ) + \overline{K}_{m}^{ ( \alpha,\beta )} \bigl( r ( t;x );x \bigr), $$

where

$$r ( t;x ) = \int_{x}^{t} ( t - u ) \bigl[ g'' ( t ) - g'' ( u ) \bigr]\,du. $$

From the mean value theorem, it follows that there exists $\xi \in ( \min ( x,t ),\max ( x,t ) )$ such that

$$r ( t;x ) = \bigl[ g'' ( x ) - g'' ( \xi ) \bigr] \int_{x}^{t} ( t - u )\,du = \bigl[ g'' ( x ) - g'' ( \xi ) \bigr]\frac{ ( t - x )^{2}}{2}. $$

So,

3.14 $$\begin{aligned} \bigl\vert r ( t;x ) \bigr\vert \le& \omega \bigl( g''; \vert t - x \vert \bigr)\frac{ ( t - x )^{2}}{2} \\ \le& \bigl( 1 + \sqrt{m + \beta + 1} \vert t - x \vert \bigr)\omega \biggl( g''; \frac{1}{\sqrt{m + \beta + 1}} \biggr)\frac{ ( t - x )^{2}}{2}. \end{aligned}$$

When $x \in [0,1]$, an upper bound for $a ( x;m )$ and $a ( 1 - x;m )$ is

3.15 $$ \bigl\vert a ( x;m ) \bigr\vert = \bigl\vert a_{1} ( m )x + a_{0} ( m ) \bigr\vert = \biggl\vert - 2 ( \alpha + 1 )x + \frac{2\alpha + 3}{2} \biggr\vert \le \frac{2\alpha + 3}{2}. $$

From (3.15) and (3.1), we get

3.16 $$ \bigl\vert \overline{K}_{m}^{ ( \alpha,\beta )} \bigl( r ( t;x );x \bigr) \bigr\vert \le \frac{2\alpha + 3}{2} \bigl\vert K_{m - 1}^{ ( \alpha,\beta + 1 )} \bigl( r ( t;x );x \bigr) + K_{m - 1}^{ ( \alpha + 1,\beta + 1 )} \bigl( r ( t;x );x \bigr) \bigr\vert . $$

Using (3.14), it follows that

3.17 $$\begin{aligned} &\bigl\vert K_{m - 1}^{ ( \alpha,\beta + 1 )} \bigl( r ( t;x );x \bigr) \bigr\vert \\ &\quad \le K_{m - 1}^{ ( \alpha,\beta + 1 )} \biggl( \bigl( 1 + \sqrt{m + \beta + 1} \vert t - x \vert \bigr)\omega \biggl( g'';\frac{1}{\sqrt{m + \beta + 1}} \biggr)\frac{ ( t - x )^{2}}{2};x \biggr) \\ &\quad \le \frac{1}{2}\omega \biggl( g''; \frac{1}{\sqrt{m + \beta + 1}} \biggr) \bigl[ K_{m - 1}^{ ( \alpha,\beta + 1 )} \bigl( ( t - x )^{2};x \bigr) \\ &\qquad {}+ \sqrt{m + \beta + 1} K_{m - 1}^{ ( \alpha,\beta + 1 )} \bigl( \vert t - x \vert ( t - x )^{2};x \bigr) \bigr]. \end{aligned}$$

Applying Corollary 2.2, it follows that there exists a constant $C'$ independent of *m* and *x* such that (3.17) becomes

3.18 $$ \bigl\vert K_{m - 1}^{ ( \alpha,\beta + 1 )} \bigl( r ( t;x );x \bigr) \bigr\vert \le C'\frac{1}{m - 1}\omega \biggl( g''; \frac{1}{\sqrt{m + \beta + 1}} \biggr). $$

Thus,

3.19 $$ \bigl\vert \overline{K}_{m}^{ ( \alpha,\beta )} \bigl( r ( t;x );x \bigr) \bigr\vert \le C\frac{1}{m}\omega \biggl( g''; \frac{1}{\sqrt{m + \beta + 1}} \biggr), $$

and the proof is completed. □

### Corollary 3.10

*For* $g \in C^{2} ( [0,1] )$, $x \in [0,1]$ *fixed*, *we have*

3.20 $$ \lim_{m \to \infty} m \bigl( \overline{K}_{m}^{ ( \alpha,\beta )} ( g;x ) - g ( x ) \bigr) = \frac{1}{2}x ( 1 - x )g'' ( x ). $$

### Proof

By Theorem 3.9 and Lemma 3.2(ii), we obtain (3.20). □

### Corollary 3.11

*For* $g \in C^{2} ( [0,1] )$, *the following estimate holds*:

3.21 $$ \bigl\Vert \overline{K}_{m}^{ ( \alpha,\beta )}g - g \bigr\Vert \le \frac{C}{m} \bigl\Vert g'' \bigr\Vert , $$

*where* $\Vert \cdot \Vert $ *is the uniform norm on* $[0,1]$.

### Proof

Since $\omega ( g'';\delta ) \le 2 \Vert g'' \Vert $, by Lemma 3.2(ii) and Theorem 3.9, we obtain (3.21). □

We can reformulate Theorem 3.9 in terms of second order moduli of continuity.

### Theorem 3.12

*Assuming* $\beta = 2\alpha$, *for* $a_{0} ( m ) = \frac{2\alpha + 3}{2}$, $a_{1} ( m ) = - 2 ( \alpha + 1 )$, *and* $g \in C ( [0,1] )$, *we have*

3.22 $$ \bigl\Vert \overline{K}_{m}^{ ( \alpha,\beta )}g - g \bigr\Vert \le C \omega_{2} \biggl( g;\frac{1}{\sqrt{m}} \biggr). $$

### Proof

The operators $\overline{K}_{m}^{ ( \alpha,\beta )}$are bounded, and by (3.1), we have

$$\bigl\Vert \overline{K}_{m}^{ ( \alpha,\beta )}g \bigr\Vert \le \bigl( \bigl\vert a_{0} ( m ) \bigr\vert + \bigl\vert a_{1} ( m ) \bigr\vert \bigr) \Vert g \Vert . $$

It is well known that the second order modulus of continuity is equivalent to the *K*-functional

$$K_{2} \bigl( g,t^{2} \bigr) = \inf_{h \in C^{2} ( [0,1] )} \bigl\{ \Vert g - h \Vert + t^{2} \bigl\Vert h'' \bigr\Vert \bigr\} . $$

From Gonska ([6], Corollary 2.7),

$$K_{2} \bigl( g,t^{2} \bigr) \le \frac{7}{2} \omega_{2} ( g,t ),\quad t \ge 0, g \in C \bigl( [0,1] \bigr). $$

Combining the above inequalities and taking the infimum over all $h \in C^{2} ( [0,1] )$ in the following inequality

$$\begin{aligned} \bigl\Vert \overline{K}_{m}^{ ( \alpha,\beta )}g - g \bigr\Vert &\le \bigl\Vert \overline{K}_{m}^{ ( \alpha,\beta )} ( g - h ) - ( g - h ) \bigr\Vert + \bigl\Vert \overline{K}_{m}^{ ( \alpha,\beta )}h - h \bigr\Vert \\ &\le C_{1} \Vert g - h \Vert + \frac{C_{2}}{m} \bigl\Vert g'' \bigr\Vert \le C_{3} \biggl\{ \Vert g - h \Vert + \frac{1}{m} \bigl\Vert g'' \bigr\Vert \biggr\} \end{aligned}$$

leads to the desired result. □

## Conclusions

In this paper, we introduce and study a modified form of the Kantorovich–Stancu operators.
